# Supplementary material for: A nanobody specific to prefusion glycoprotein B neutralizes HSV-1 and HSV-2
Source: Nature. 2025 Sep 3;646(8084):433–41. doi: 10.1038/s41586-025-09438-5 (PMC12507662; doi:10.1038/s41586-025-09438-5)
Supplement: Supplementary file 1 — Supplementary Figs. 1–11 and Discussion. Figs. 1–7 provide details on protein purifications, electron microscopy density fitting and structure alignment, affinity measurements and Nb1_gbHSV epitope conservation. The discussion analyses and compares the fusion mechanism with other class III viral fusion proteins (Figs. 8 and 9). Figs. 10 and 11 contain uncut blots and gels. [file 41586_2025_9438_MOESM1_ESM.pdf]

nature portfolio

https://doi.org/10.1038/s41586-025-09438-5

# Supplementary information

# A nanobody specific to prefusion glycoprotein B neutralizes HSV-1 and HSV-2

In the format provided by the authors and unedited

# Supplementary information

## A nanobody specific to prefusion glycoprotein B neutralises HSV-1 and -2

Benjamin Vollmer [1,2,3], Henriette Ebel [1,2,3], Renate Rees [4], Julia Nentwig [1,2,3], Thomas Mulvaney [1,5,6], Jürgen Schünemann [4], Jens Krull [4], Maya Topf [1,5,6], Dirk Görlich [4*], Kay Grünewald [1,2,3*]

**Affiliation**
[1]Centre for Structural Systems Biology (CSSB), Hamburg, Germany
[2]Department of Chemistry, University of Hamburg, Hamburg, Germany
[3]Department of Structural Cell Biology of Viruses, Leibniz Institute of Virology (LIV), Hamburg, Germany
[4]Department of Cellular Logistics, Max Planck Institute for Multidisciplinary Sciences, Göttingen, Germany
[5]University Medical Center Hamburg-Eppendorf (UKE), Hamburg, Germany
[6]Department of Integrative Virology, Leibniz Institute of Virology (LIV), Hamburg, Germany

*co-corresponding authors: goerlich@mpinat.mpg.de, kay.gruenewald@cssb-hamburg.de

Table of contents
Supplementary Figures S1-S7 ........................................ 2-10
Supplementary Discussion with Figures S8-S9 ........................................ 11-13
Supplementary References ........................................ 13
Supplementary Figures S10-S11 (uncropped gels and blots) ........................................ 14-15

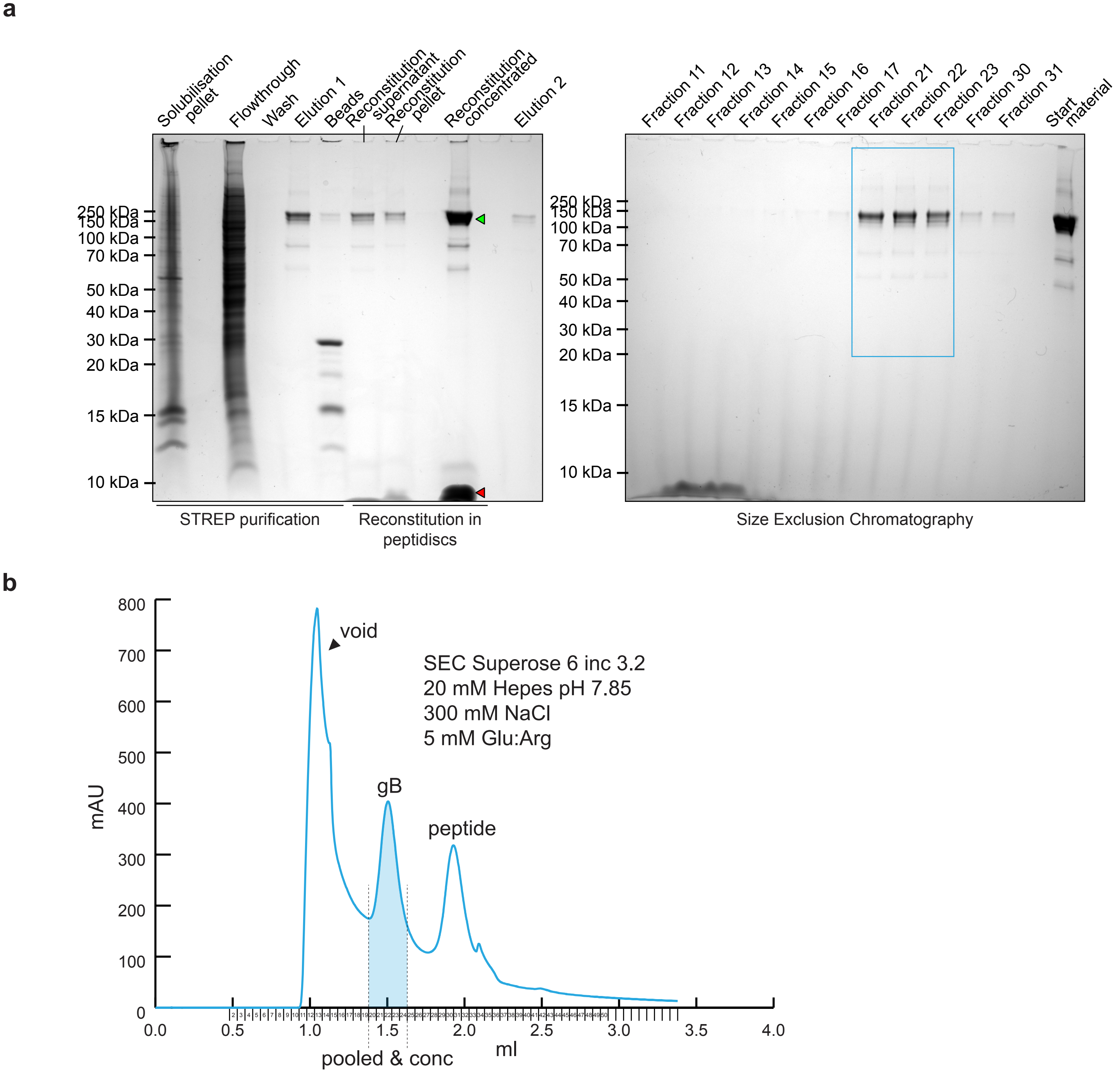


**Supplementary Figure S1 - Stabilised gB purification & reconstitution**

**a** SDS PAGE analysis of the individual purification steps including affinity chromatography via the Twin-Strep-tag (STREP), reconstitution in peptidiscs and SEC. The green arrowhead marks the gB band, the red arrowhead marks the peptidisc peptide band. The starting material and individual peak fractions are shown on the right with fractions that were pooled, concentrated and used for CryoEM labelled by the blue box. The corresponding SEC profile is shown in **b**. Marked are peaks of the void, gB and the excess peptidisc peptide that was not incorporated during reconstitution. For gel source data, see Supplementary Figure S11.

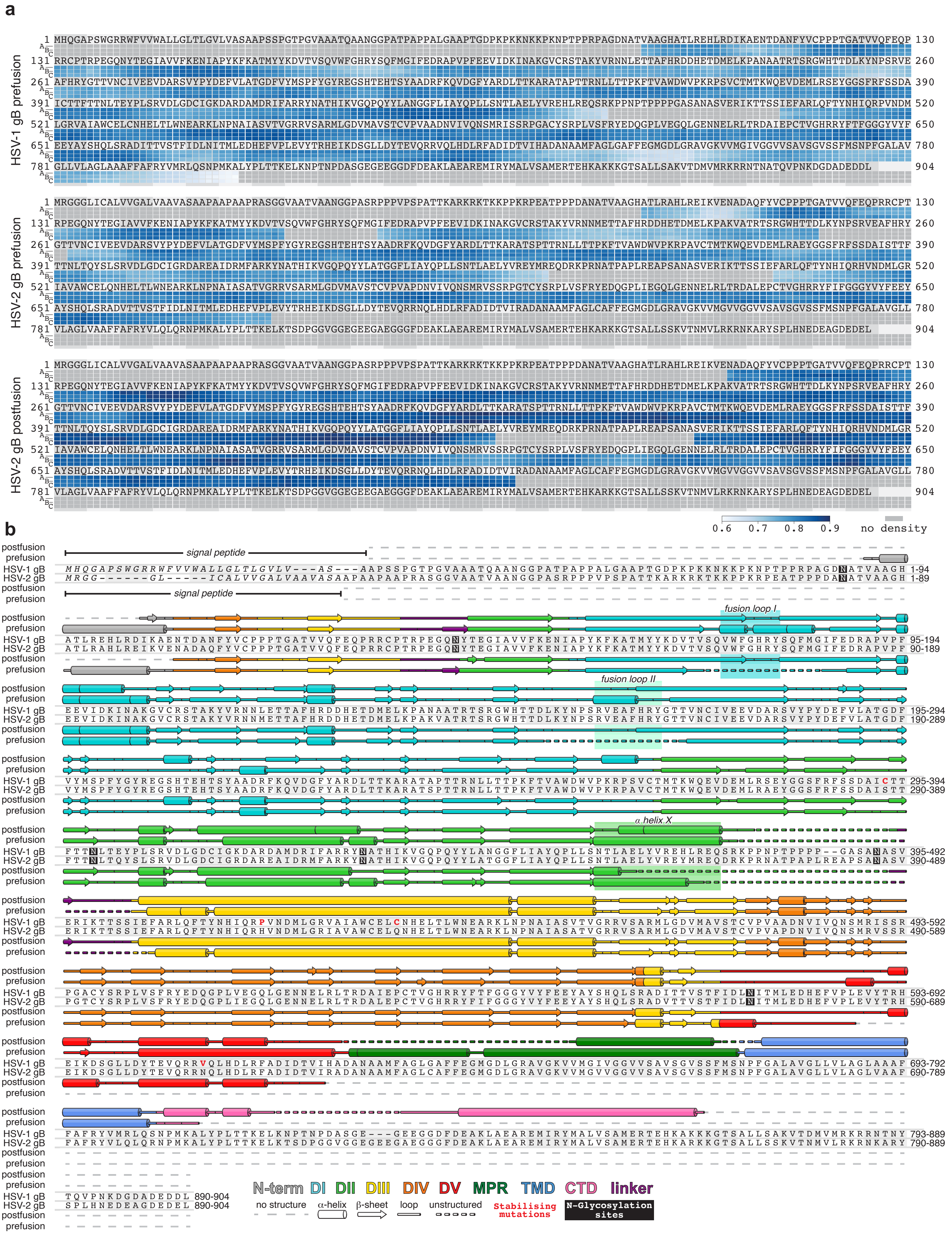
a
HSV-1 gB prefusion
HSV-2 gB prefusion
HSV-2 gB postfusion
0.6 0.7 0.8 0.9
no density
b
signal peptide
fusion loop I
fusion loop II
α helix X
postfusion
prefusion
HSV-1 gB
HSV-2 gB
N-term DI DII DIII DIV DV MPR TMD CTD linker
no structure
α-helix
β-sheet
loop
unstructured
Stabilising mutations
N-Glycosylation sites

**Supplementary Figure S2 - Manders' Overlap Coefficient (SMOC) analysis and structural alignment**
**a** SMOC analysis showing a sequence-based local estimate of the fit quality of the atomic model to the corresponding map (0 – 1, meaning no fit to perfect fit) for every amino acid of each chain of each protein[68]. To emphasize the differences in the calculated range, only values ranging from 0.6 to 0.9 are depicted as shades of blue. Unmodeled parts of the structure which have no SMOC score, are shown in grey.
**b** Structure alignment analysis showing secondary structure elements of gB from HSV-1 in post- (PDB: 5V2S)[3] and prefusion (Nb1_gbHSV bound) conformation and of HSV-2 in post- and prefusion form. Domain boundaries are shown by colours as in Fig. 2. N-glycosylation sites are shown with black background, while stabilising mutations used in HSV-1 prefusion gB are shown in bold red letters. Regions comprising the fusion loops as well as $\alpha$-helix X are marked. Given the symmetric nature of the pre and post fusion trimer only the secondary structure for chain A is plotted.

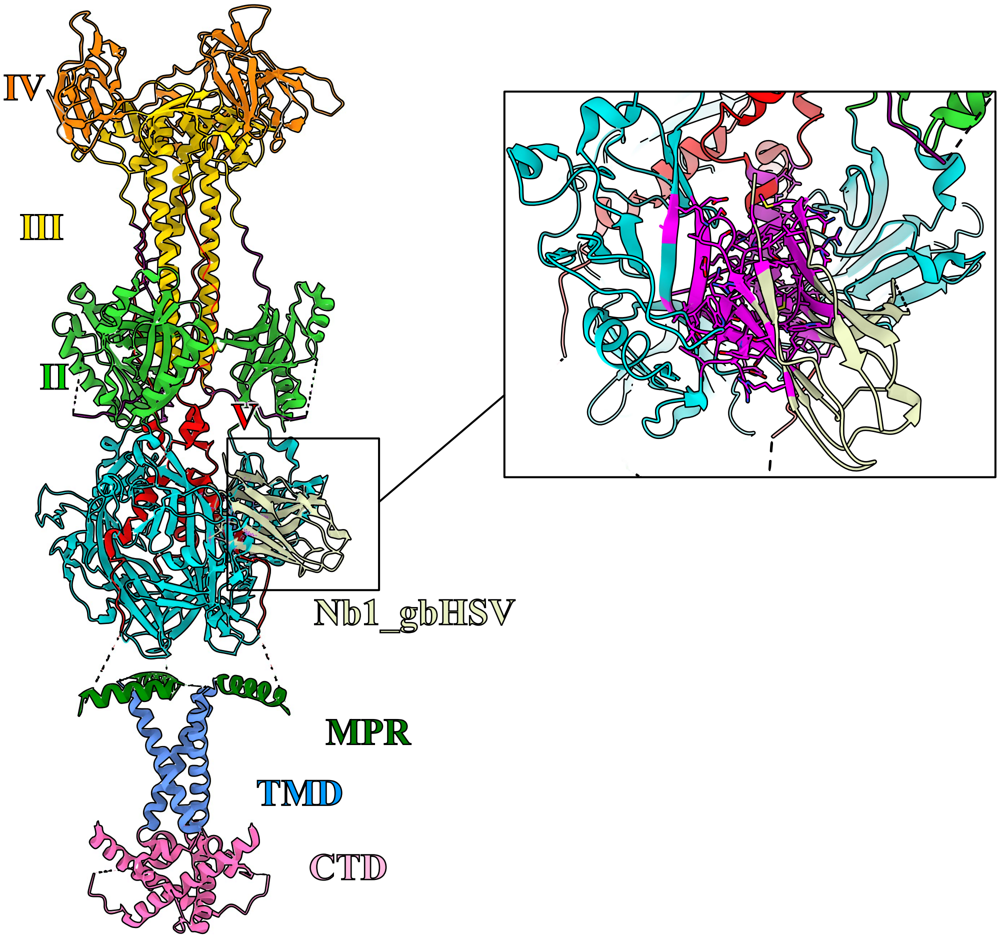


**Supplementary Figure S3 - Clash analysis of Nb1_gbHSV on postfusion gB**
Mapping of Nb1_gbHSV onto the postfusion structure (PDB: 5V2S), bound to its main interaction surface on DI revealed 1029 clashes, mainly with DV and DI of the neighbouring protomer. Domains are coloured as in Fig. 2. Residues involved in clashes (VDW overlap > 0.6 Å) are coloured magenta in the zoom-in inset.

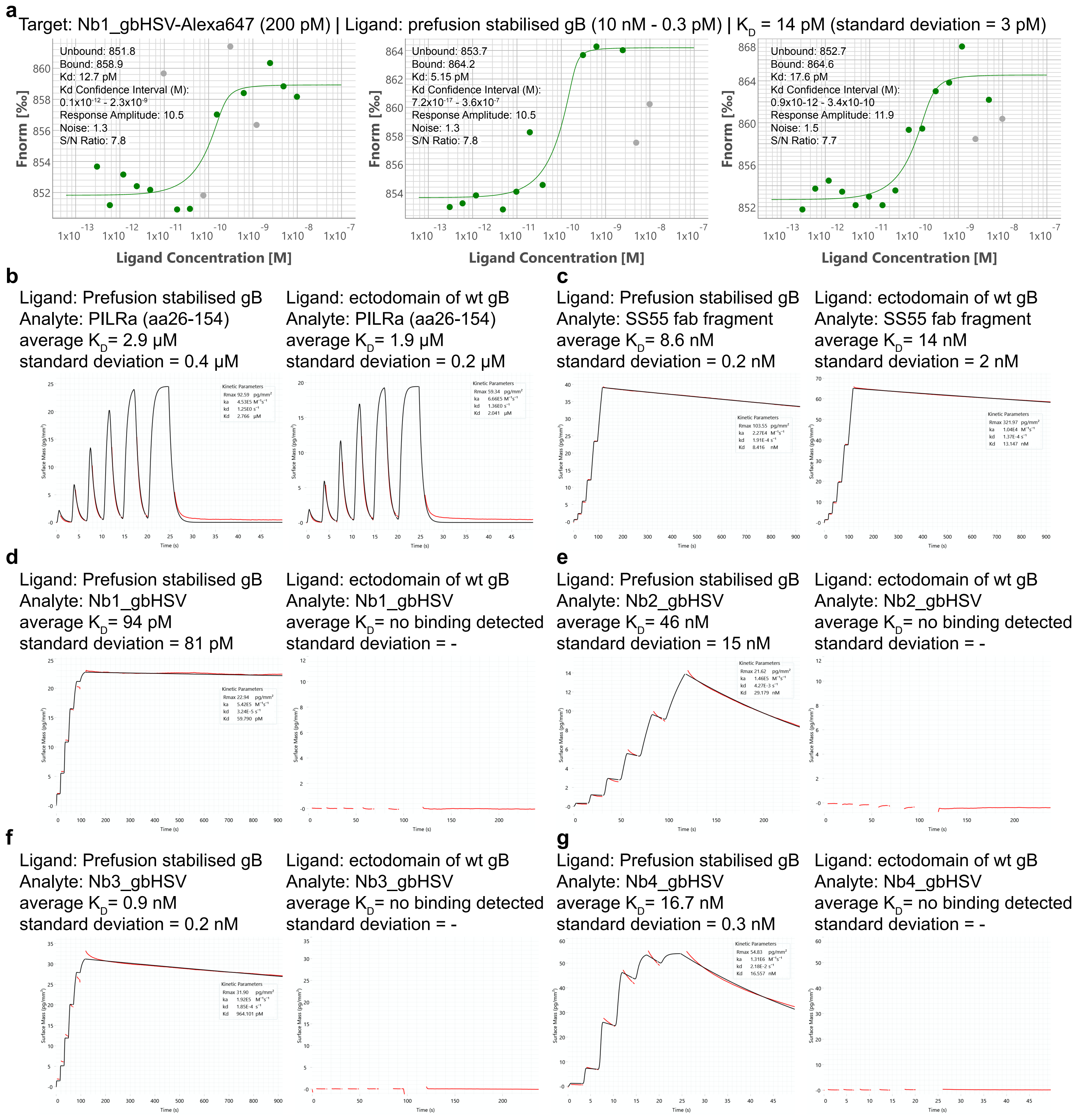


**Supplementary Figure S4 – Determination of conformation specificity of nanobodies binding gB**

**a** Shown is the fit of three independent MST measurements using 200 pM Nb1_gbHSV-Alexa647 as target in a 16 point 1:1 dilution series of prefusion stabilised gB ranging from 10 nM to 0.3 pM. The reported $K_D$ is the average of three independent measurements.

**b-g** Binding affinities of PILRα (a), Fab SS55 (b) and nanobodies (c-d) to pre- and postfusion gB were measured using GCI. Prefusion-stabilised or postfusion gB (ectodomain of WT) were immobilised in respective channels and WaveRAPID measurements were conducted to determine binding affinities using the intermediate (Nb4_gbHSV, PILRα) or tight (Nb1_gbHSV, Nb2_gBHSV, Nb3_gbHSV, SS55) binder protocol. Each measurement was repeated three times to calculate the average binding affinity ($K_D$) and standard deviation and of which one representative measurement is shown.

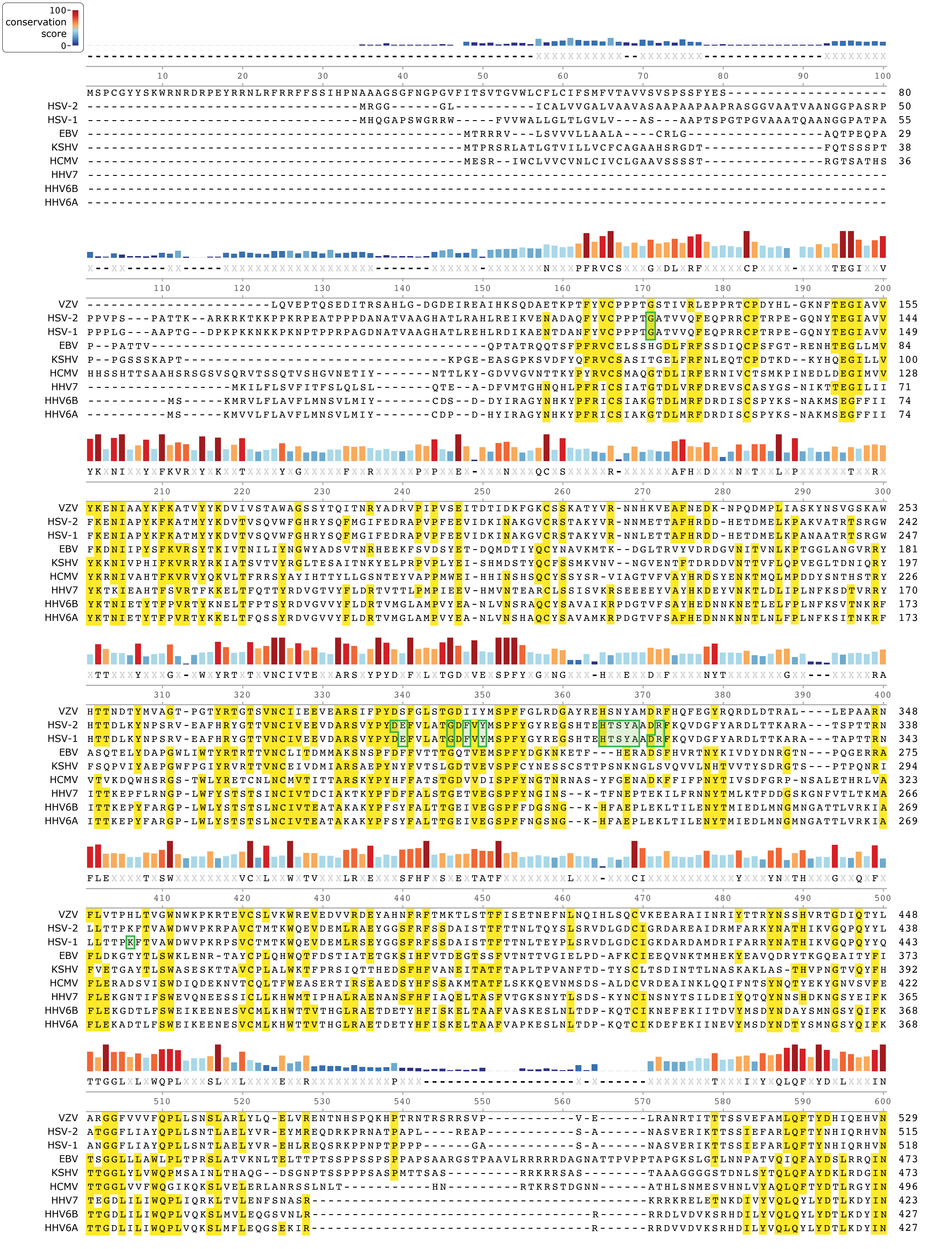
100-
conservation score
0-
10 20 30 40 50 60 70 80 90 100
VZV
HSV-2
HSV-1
EBV
KSHV
HCMV
HHV7
HHV6B
HHV6A
110 120 130 140 150 160 170 180 190 200
210 220 230 240 250 260 270 280 290 300
310 320 330 340 350 360 370 380 390 400
410 420 430 440 450 460 470 480 490 500
510 520 530 540 550 560 570 580 590 600

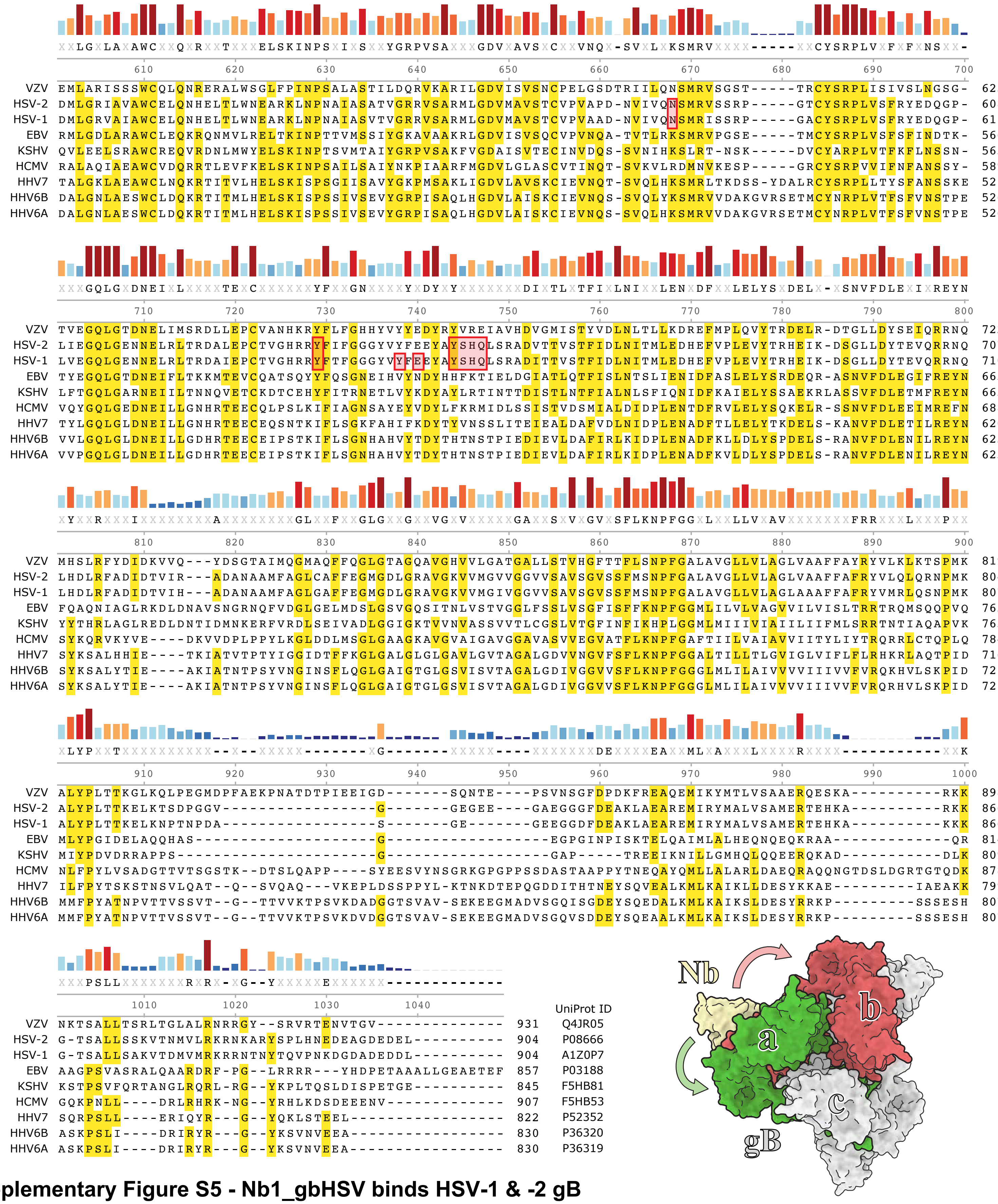


**Supplementary Figure S5 - Nb1_gbHSV binds HSV-1 & -2 gB**

Clustal Omega multiple sequence alignment. Conservation score[66,67] for each position is shown as coloured bars followed by the consensus sequence. VZV: varicella zoster virus; HSV-2: herpes simplex virus 2; HSV-1: herpes simplex virus 1; EBV: Epstein-Barr virus; KSHV: Kaposi's sarcoma-associated herpesvirus; HCMV: human. Cytomegalovirus; HHV7: human herpesvirus 7; HHV6A, B: human herpesvirus 6A, B. Residues interacting with one Nb1_gbHSV are marked with red boxes (gB protomer a) and green boxes (gB protomer b). UniProt IDs of the respective sequences are shown at the end. To illustrate the inter-protomer interaction a top view of gB is shown on the bottom right with protomer a in green and b in red and Nb1_gbHSV in antique white.

**a**

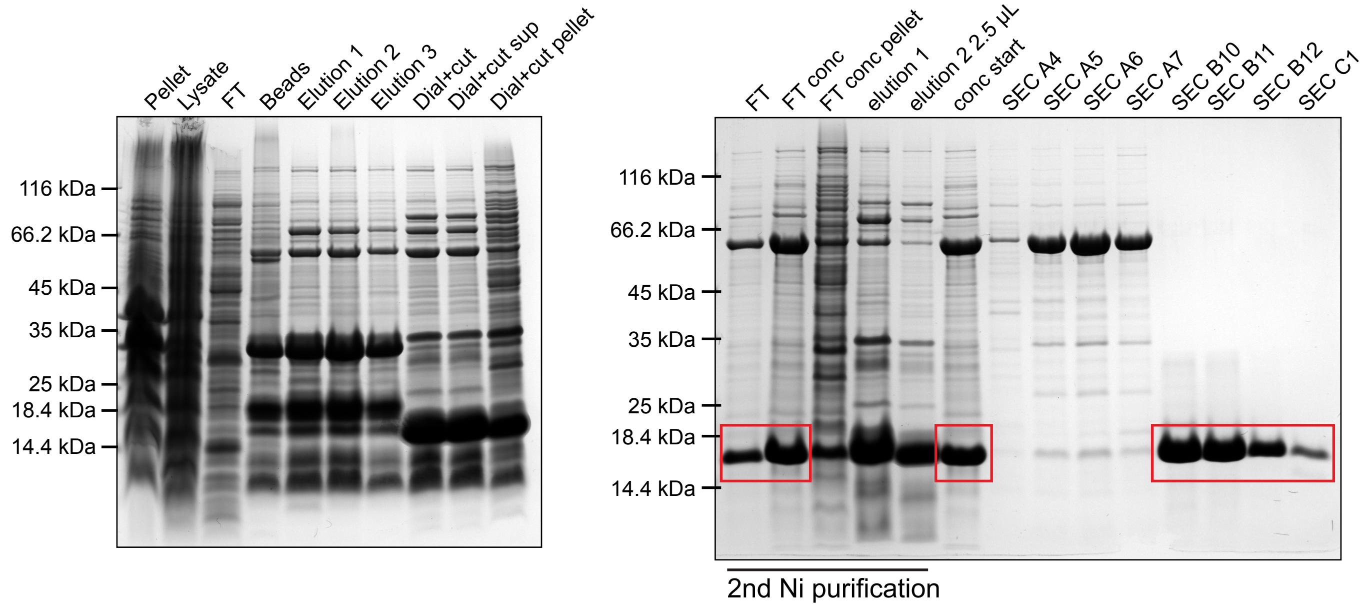


**b**

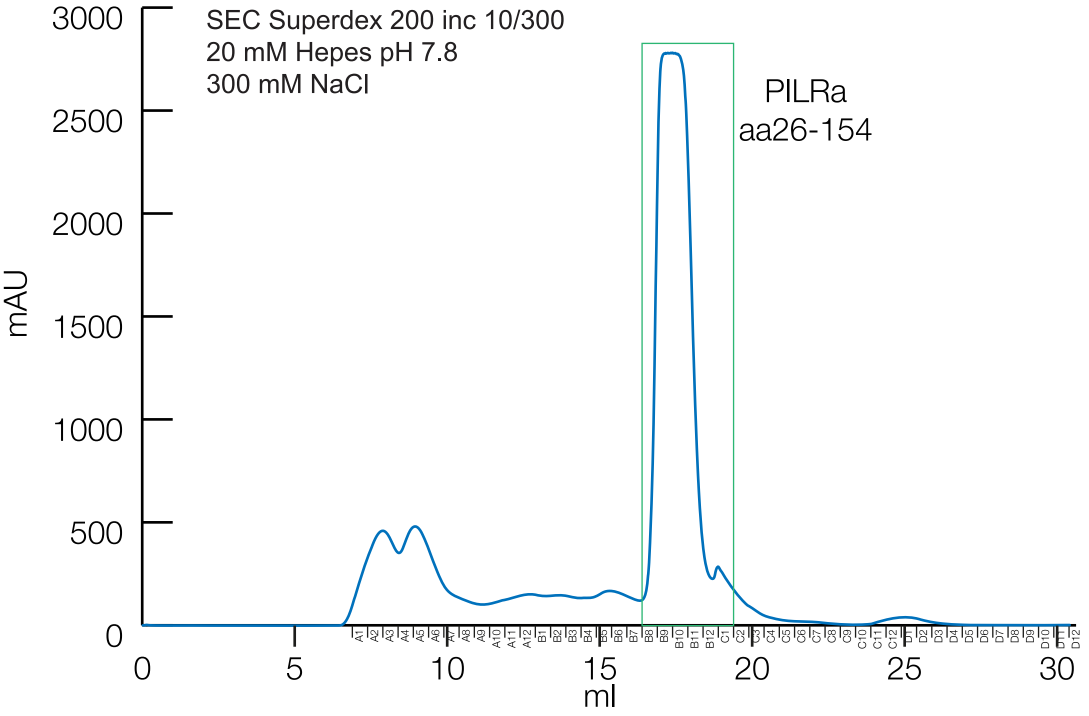


**Supplementary Figure S6 - Purification of PILRa IgV**

**a** SDS PAGE analysis of the individual purification steps including affinity chromatography via the N-terminal $His_{14}$-Tag, NEDD8 tag cleavage, rebind to NiNTA and SEC. Red boxes mark the PILRa band.

**b** SEC profile of conc. flowthrough after rebind. Fractions B8-C1 marked by green box were pooled, concentrated and stored in -70°C till being used. For gel source data, see Supplementary Figure S11.

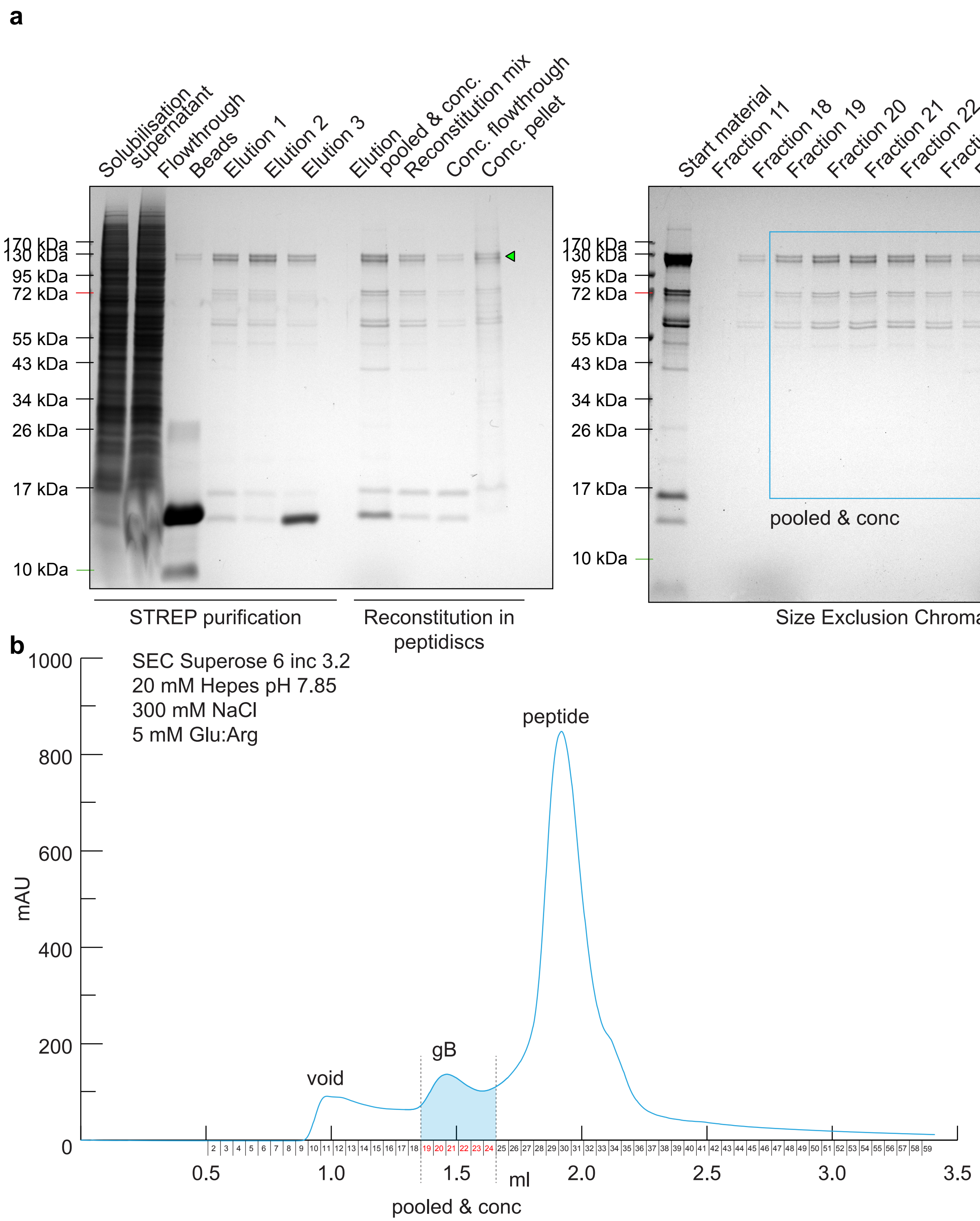


**Supplementary Figure S7 - HSV-2 gB purification & reconstitution**

**a** SDS PAGE analysis of the individual purification steps including affinity chromatography via the Twin-Strep-tag (STREP), reconstitution in peptidiscs and SEC. The green arrowhead marks the gB band. The starting material and individual peak fractions are shown on the right with fractions that were pooled, concentrated and used for CryoEM labelled by the blue box. The corresponding SEC profile is shown in **b**. Marked are peaks of the void, gB and the excess peptidisc peptide that was not incorporated during reconstitution. For gel source data, see Supplementary Figure S11.

## Supplementary Discussion

The order of events during the fusion process performed by gB can be indicatively derived by examining the changes between its pre and postfusion structures as well as those of related class III viral membrane fusion proteins. These include glycoprotein G of vesicular stomatitis virus (VSV-G) and baculovirus GP64. VSV-G protomers consist of three distinct domains, connected by five refolding segments (R1-R5), which enable the conformational changes during fusion[72,73]. These segments are also present in gB, performing a similar role (Supplementary Figure S8): R1 and R4 correspond to the linkers connecting DII to DIII (aa135-142 and aa501-510), while R2 and R3 correspond to the linkers that connect DI to DII (aa152-156 and aa356-365). R5 that describes the C-terminal part finds only partial resemblance in gB (~aa660-701).

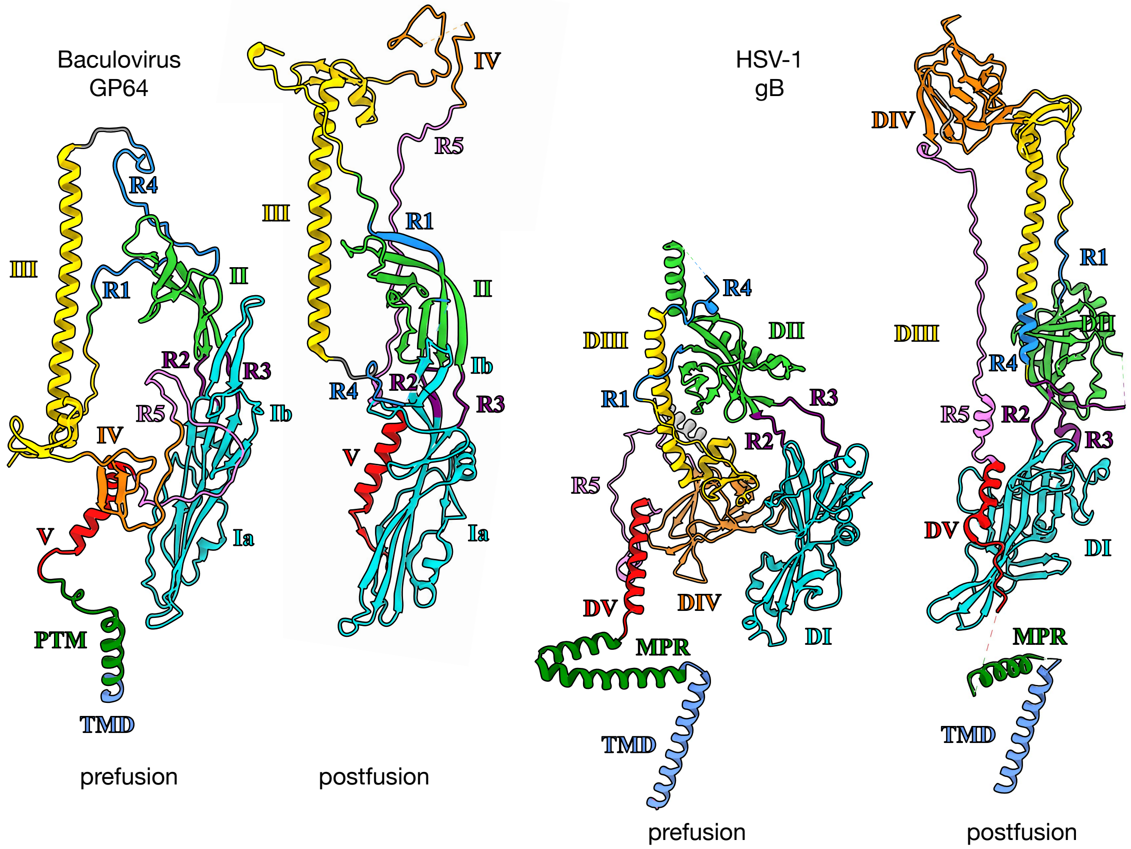


**Supplementary Figure S8**

Protomer structures are shown for Baculovirus GP64 in pre- (PDB: 8VG6) and postfusion (PDB:3DUZ) conformation and HSV-1 gB (this work and PDB:5V2S). For better comparison, corresponding domains are coloured as in Fig. 2. Refolding regions R1-R5 as defined in[72] for VSV-G and corresponding regions are shown in blue: R1 & R4 connecting domains II and III, purple: R2 & R3 connecting domains I and II; and magenta: R5 connecting domains IV and V.

The recently reported prefusion and early intermediate structures of the baculovirus GP64 protein[74] offer additional insights, beyond what was described for rhabdoviridae[72,73,75] as there are certain structural differences between these proteins. Notably, in GP64, R2 and R3 are less pronounced due to structural differences in the fusion-loop-carrying domain (Supplementary Figure S8) and R4 does not undergo the loop-to-helix transition seen in HSV-1 gB and VSV-G. R5 in GP64 and gB is part of DV that features a C-terminal helix involved in a short coiled-coil preceding the

membrane embedded parts (Supplementary Figure S8), which is not seen in prefusion VSV-G. Furthermore, the fusion process for the latter involves monomeric intermediate states[72], which is less likely for GP64 and gB as the protomers of these proteins are much more intertwined by cyclic domain swapping and additional interactions formed within the coiled-coil in DV and between the transmembrane helices[16].

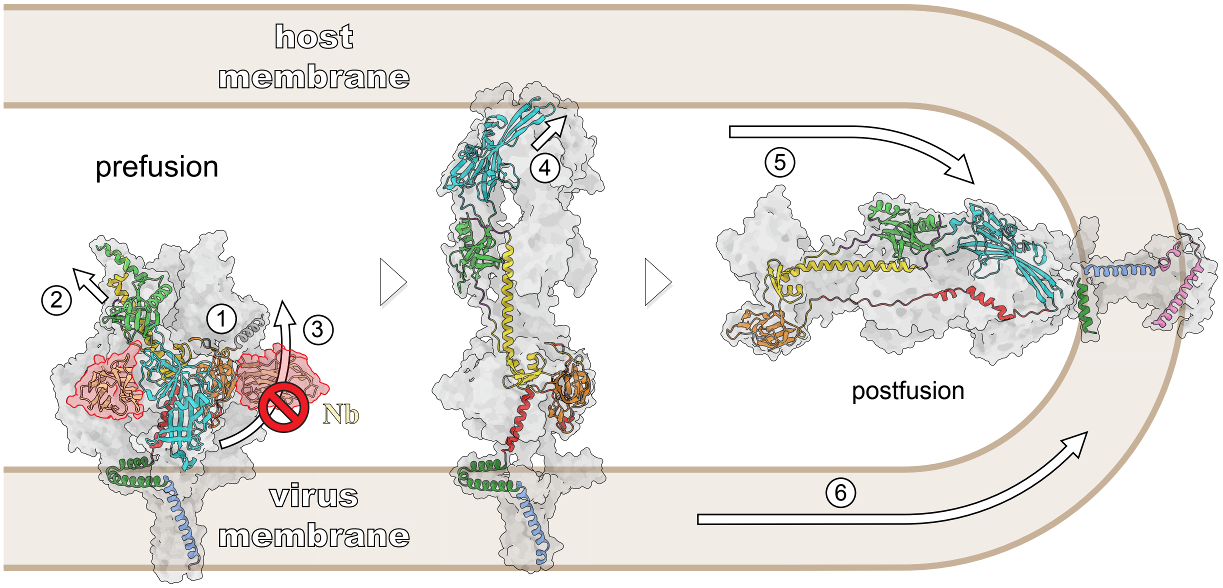


**Supplementary Figure S9**: Fusion mechanism
Proposed conformational changes of gB during the membrane fusion process. For clarity, individual gB protomers of the trimers are shown with the full trimer as semi-transparent surface rendering. The extended intermediate model in the middle is composed of residues 100-557 of the postfusion gB conformation[3] and 558-803 of the stabilised HSV-1 gB structure. The HSV-1 full-length postfusion structure (PDB: 5V2S) is shown on the right. Possible sequence of events in the fusion process (1)-(6) is described in the text.

The start of the fusion process was described for GP64 to involve rotation of DI outwards, triggered by protonation of histidine residues in a low pH environment. For gB, in the absence of a histidine cluster to trigger the fusion process, this outward movement of DI is probably only possible once the N-terminal helix of the neighbouring protomer is released (Supplementary Figure S9 (**1**)). In GP64 refolding of R1 and the subsequent loss of interactions within the trimer, allow unfolding of R4 and relaxation of the other refolding regions. These changes enable the ~180° rotation of DI and DII leading to an extended intermediate state. A similar process to reach this state can be envisioned for gB. Refolding of R1 as well as R4 on αC in DIII to extend the helix upwards (**2**) probably initiates the upwards rotation of DI and DII (**3**). This way, αX is released and flexible to loosely interact with DII. Nb1_gbHSV links DI to DIV of neighbouring protomers within the trimer (Fig. 4) and prevents gB to reach the postfusion conformation (Fig. 5) resulting in effective neutralisation (Fig. 1).
In absence of the nanobody, the fusion loops are freed from the MPR (Fig. 3) and extended from their 'rolled up' conformation into extended beta hairpins (Fig. 2), approach the target membrane and insert (**4**). This extended intermediate state of gB is probably very short lived as (**5**) via zippering down DV along the length of the molecule, the gB ectodomain adopts a hairpin prototypic for class I to III viral fusion proteins. During rearrangement of DV the membrane embedded regions pull the virus and host membrane together (**6**) and complete the fusion process. Interestingly, in a recently published preprint[43], yet another order of events has been proposed, inferred from different ectodomain constructs of EBV gB, trapped in what are assumed to be

different states of the pre- to postfusion transition. The authors suggest that refolding of DV along the central helix happens much earlier in the process in order to drive the reorientation of DI towards the host cell membrane. The extended intermediate state would therefore have DV already in postfusion conformation, while the final pull of the membrane would be performed by flipping and re-association of DII with the rest of gB. This alternative model presents an intriguing new perspective, which aligns more with what has been proposed for vesiculoviruses, where R5, after being freed from the fusion domain (DI), refolds to initiate the subsequent conformational changes[72]. However, just as class III viral fusion proteins exhibit diverse architectures, their fusion processes may also vary among different members and further research is necessary to thoroughly validate the unique steps involved in each case.

## Supplementary References


72 Baquero, E. et al. Structural intermediates in the fusion-associated transition of vesiculovirus glycoprotein. EMBO J **36**, 679-692, doi:10.15252/embj.201694565 (2017).

73 Beilstein, F. et al. Identification of a pH-Sensitive Switch in VSV-G and a Crystal Structure of the G Pre-fusion State Highlight the VSV-G Structural Transition Pathway. Cell Rep **32**, 108042, doi:10.1016/j.celrep.2020.108042 (2020).

74 Guo, J. et al. Structural transition of GP64 triggered by a pH-sensitive multi-histidine switch. Nat Commun **15,** 7668, doi:10.1038/s41467-024-51799-4 (2024).

75 Yang, F. et al. Structural Analysis of Rabies Virus Glycoprotein Reveals pH-Dependent Conformational Changes and Interactions with a Neutralizing Antibody. Cell Host Microbe **27**, 441-453 e447, doi:10.1016/j.chom.2019.12.012 (2020).

**a** For Extended Data Fig. 1d

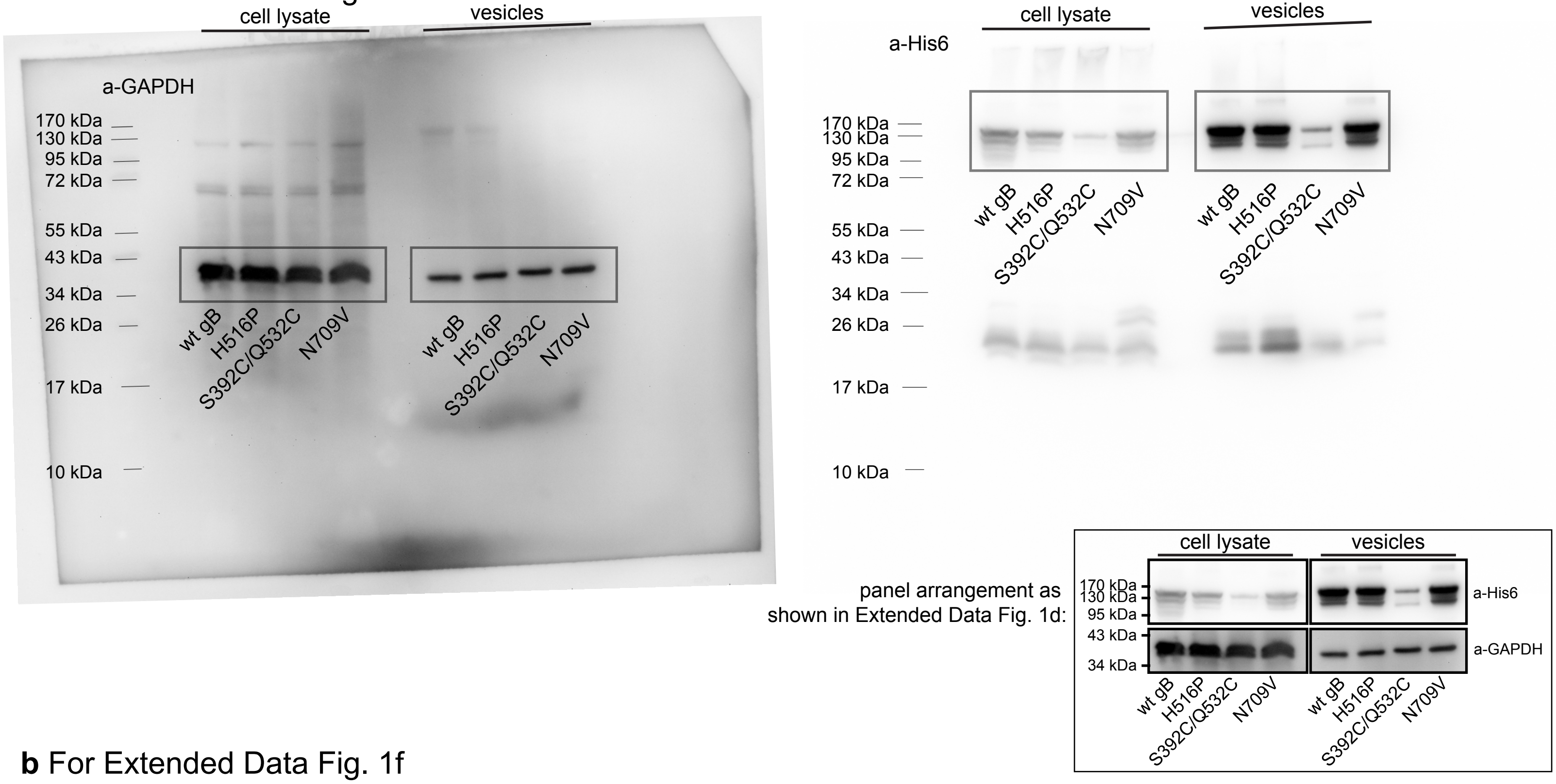


**b** For Extended Data Fig. 1f

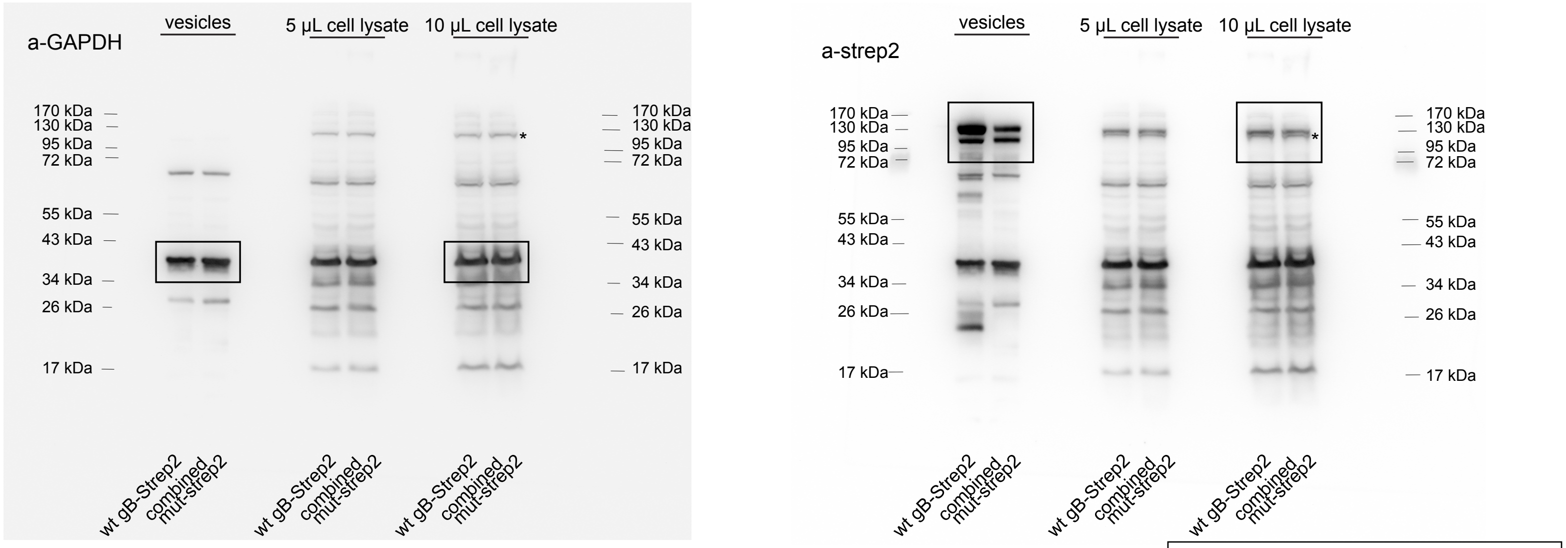


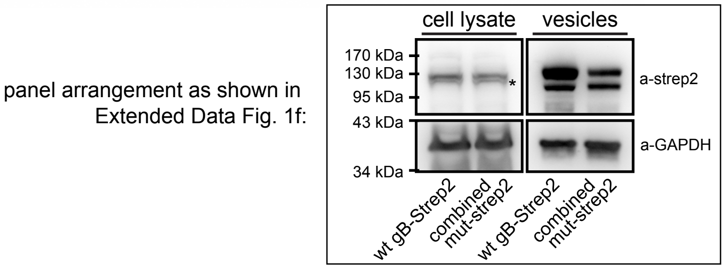


**Supplementary Figure S10**

Uncropped images of Western Blots shown in Extended Data Figure 1d and 1f. Boxes mark the shown selection/cropping for the final figure which is shown below respectively.

**a** All samples were run on the same gel. After SDS-PAGE samples were transferred on a blot which was stained sequentially. To visualise the loading control first a 1:2500 dilution of mouse $\alpha$-GAPDH was used followed by a 1:5000 dilution of secondary $\alpha$-mouse-HRP. After exposure and extensive washing a 1:5000 dilution of rabbit $\alpha$-His6 antibody followed by a 1:5000 dilution of secondary $\alpha$-rabbit-HRP was used to detect the proteins of interest.

**b** Done as described in **a**, except to detect the proteins of interest a 1:4000 dilution of Strep-Tactin-HRP was used. Please note, in order to keep the order of samples as for Extended Data Fig, 1d, cell lysate bands are shown before vesicle bands in Extended Data Fig. 1f. Unspecific band in strep blot panel originating from the $\alpha$GAPDH antibody is marked by an asterisk.

For Supplementary Fig. S1

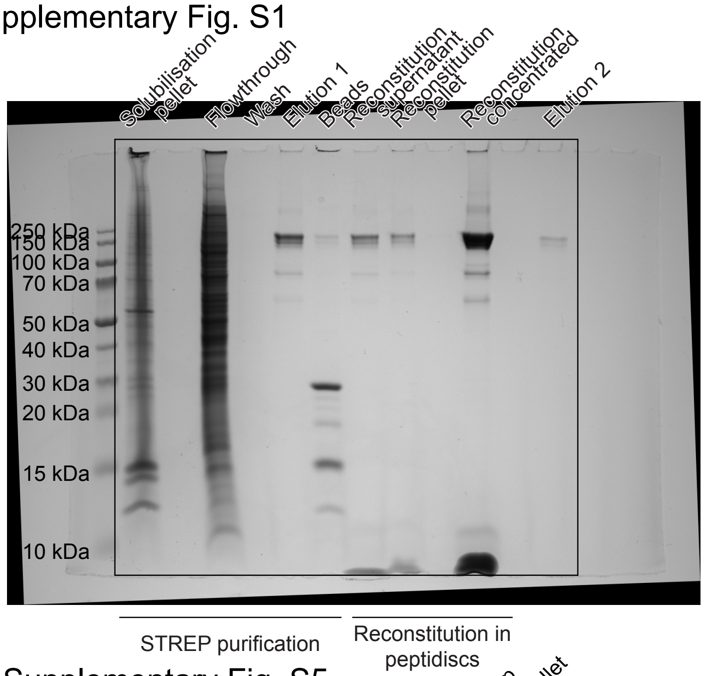


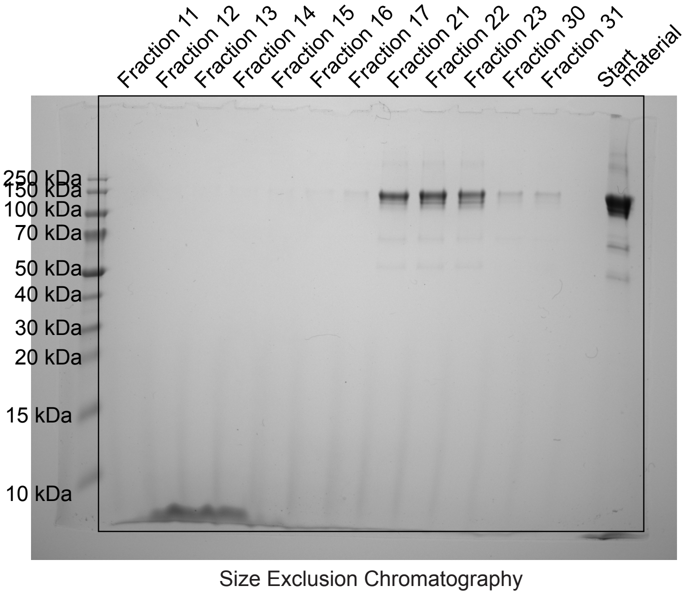


For Supplementary Fig. S5

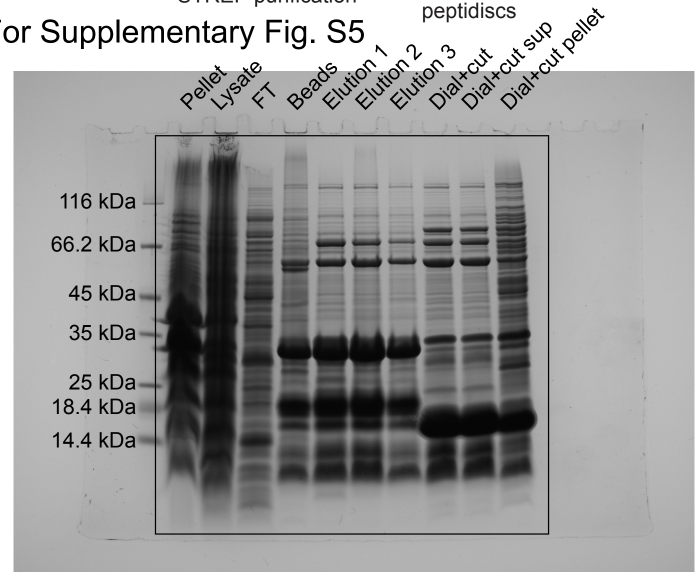


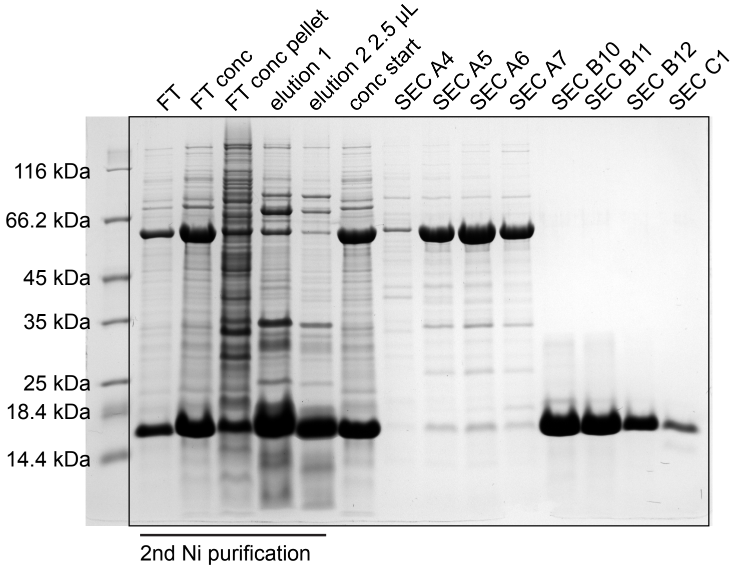


For Supplementary Fig. S7

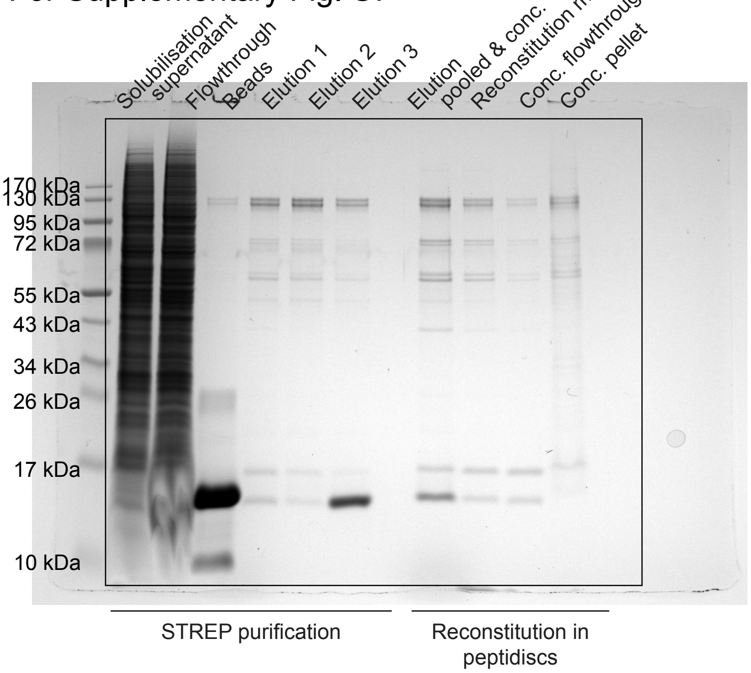


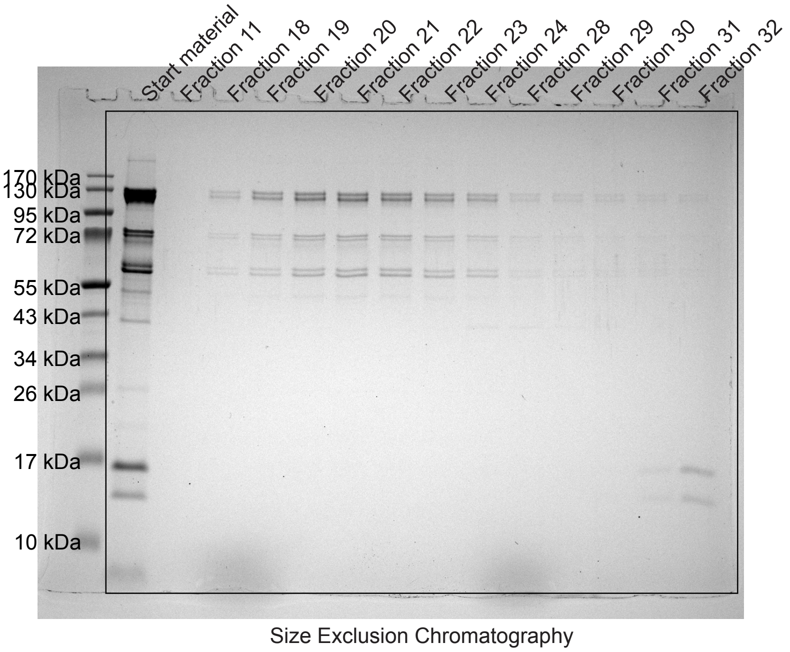


**Supplementary Figure S11**
Uncropped images of SDS PAGE gels shown in Supplementary Material. Boxes mark the shown selection in the final figure.
