# Supplementary material for: A nanobody specific to prefusion glycoprotein B neutralizes HSV-1 and HSV-2
Source: Nature. 2025 Sep 3;646(8084):433–41. doi: 10.1038/s41586-025-09438-5 (PMC12507662; doi:10.1038/s41586-025-09438-5)
Supplement: Supplementary file 2 — Reporting Summary [file 41586_2025_9438_MOESM2_ESM.pdf]

nature portfolio

Corresponding author(s): Gruünewald, K.

Last updated by author(s): Jul 19, 2025

Reporting Summary

Nature Portfolio wishes to improve the reproducibility of the work that we publish. This form provides structure for consistency and transparency in reporting. For further information on Nature Portfolio policies, see our Editorial Policies and the Editorial Policy Checklist.

Statistics

For all statistical analyses, confirm that the following items are present in the figure legend, table legend, main text, or Methods section.

n/a	Confirmed	
☐	☒	The exact sample size (*n*) for each experimental group/condition, given as a discrete number and unit of measurement
☐	☒	A statement on whether measurements were taken from distinct samples or whether the same sample was measured repeatedly
☒	☐	The statistical test(s) used AND whether they are one- or two-sided<br>*Only common tests should be described solely by name; describe more complex techniques in the Methods section.*
☒	☐	A description of all covariates tested
☒	☐	A description of any assumptions or corrections, such as tests of normality and adjustment for multiple comparisons
☐	☒	A full description of the statistical parameters including central tendency (e.g. means) or other basic estimates (e.g. regression coefficient) AND variation (e.g. standard deviation) or associated estimates of uncertainty (e.g. confidence intervals)
☒	☐	For null hypothesis testing, the test statistic (e.g. *F*, *t*, *r*) with confidence intervals, effect sizes, degrees of freedom and *P* value noted<br>*Give P values as exact values whenever suitable.*
☒	☐	For Bayesian analysis, information on the choice of priors and Markov chain Monte Carlo settings
☒	☐	For hierarchical and complex designs, identification of the appropriate level for tests and full reporting of outcomes
☒	☐	Estimates of effect sizes (e.g. Cohen's *d*, Pearson's *r*), indicating how they were calculated

Our web collection on statistics for biologists contains articles on many of the points above.

Software and code

Policy information about availability of computer code

Data collection	SerialEM 4.0
Data analysis	Excel 16.16.27(201012) (Microsoft), CryoSPARCv4, WARP (http://www.warpem.com/warp/), ISOLDE 1.8, ChimeraX 1.8, Phenix version 1.20.1-4487, MolProbity 4.5.2, CCP-EM Doppio 0.5.0, coot 0.9.8.93, TEMPyReFF, WAVEcontrol software (Creoptix AG), MO.control (Nanotemper), SnapGene v8.1, Adobe Illustrator 2024, Adobe Photoshop 2024

For manuscripts utilizing custom algorithms or software that are central to the research but not yet described in published literature, software must be made available to editors and reviewers. We strongly encourage code deposition in a community repository (e.g. GitHub). See the Nature Portfolio guidelines for submitting code & software for further information.

Data

Policy information about availability of data

All manuscripts must include a data availability statement. This statement should provide the following information, where applicable:

- Accession codes, unique identifiers, or web links for publicly available datasets
- A description of any restrictions on data availability
- For clinical datasets or third party data, please ensure that the statement adheres to our policy

The EM density maps have been deposited in the Electron Microscopy Data Bank under EMD-52963, EMD-52965, EMD-52863 and EMD-52966. The corresponding models have been deposited in the Protein Data Bank under 9Q9L, 9Q9N, 9IH8, 9Q9S.

In preparation of this manuscript the following, atomic structures have been used that are available on the Protein Data Bank: 1IGT, 6CGR, 6Z9M, 5V2S, 2GUM, 7KDP, 8VG6, 3DUZ. For sequence alignments the following sequences were used that are available on the UniProt database: Q4JR05, P08666, A1Z0P7, P03188, F5HB81, F5HB53, P52352, P36320, P36319.

Research involving human participants, their data, or biological material

Policy information about studies with human participants or human data. See also policy information about sex, gender (identity/presentation), and sexual orientation and race, ethnicity and racism.

Reporting on sex and gender	n/a
Reporting on race, ethnicity, or other socially relevant groupings	n/a
Population characteristics	n/a
Recruitment	n/a
Ethics oversight	n/a

Note that full information on the approval of the study protocol must also be provided in the manuscript.

Field-specific reporting

Please select the one below that is the best fit for your research. If you are not sure, read the appropriate sections before making your selection.

☒ Life sciences ☐ Behavioural & social sciences ☐ Ecological, evolutionary & environmental sciences

For a reference copy of the document with all sections, see nature.com/documents/nr-reporting-summary-flat.pdf

Life sciences study design

All studies must disclose on these points even when the disclosure is negative.

Sample size	No statistical methods were used to predetermine sample size. The data size for cryoEM was determined by the availability of the microscope time and the particle density on the grids. Sufficient cryo-EM data were collected to achieve the reported resolution of map, which is sufficient for model building. The sample sizes for functional assays were chosen to ensure reproducibility.
Data exclusions	CryoEM data processing involved removing poor-quality or damaged particles to achieve high resolution maps through pre-established standard data classification procedures. No other data was excluded.
Replication	Screening of HSV-1 neutralisation by different nanobodies was done with at least two successful, technical replicates. IC50 value for Nb1_gbHSV was determined from three successful biological replicates with three technical replicates each. Expression analysis for disulfide constructs was determined from three successful biological replicates. Expression and protein conformation analysis of different stabilised constructs of gB is shown by Western Blot analysis and by cryoET as examples of one of at least two successful biological replicates. Binding tests of Nb1_gbHSV to different homologues by cell surface staining of gB was analysed from three successful biological replicates. Affinity measurements of the different nanobodies or controls to gB were determined each from three successful biological replicates.
Randomization	This is not relevant to our study, because no grouping was needed. Only during cryoEM data analysis randomisation is used, which is automatically applied by the used software.
Blinding	Investigators were not blinded to group allocation, because no grouping was needed for this study. Only during cryoEM data analysis 'blinding' was used by omitting the use of preliminary models and instead using ab-inito models generated by the data itself for all structures.

Reporting for specific materials, systems and methods

We require information from authors about some types of materials, experimental systems and methods used in many studies. Here, indicate whether each material, system or method listed is relevant to your study. If you are not sure if a list item applies to your research, read the appropriate section before selecting a response.

Materials & experimental systems

n/a	Involved in the study	
☐	☒	Antibodies
☐	☒	Eukaryotic cell lines
☒	☐	Palaeontology and archaeology
☐	☒	Animals and other organisms
☒	☐	Clinical data
☒	☐	Dual use research of concern
☒	☐	Plants

Methods

n/a	Involved in the study	
☒	☐	ChIP-seq
☒	☐	Flow cytometry
☒	☐	MRI-based neuroimaging

Antibodies

Antibodies used	rabbit anti-His6 antibody (abcam, ab9108), anti-rabbit-HRP (Sigma-Aldrich Chemie GmbH, A0545), mouse anti-GAPDH antibody (Sigma-Aldrich Chemie GmbH, G8795), anti-mouse-HRP (Sigma-Aldrich Chemie GmbH, AP181P), Strep-Tactin® HRP (iba Lifesciences, 2-1502-001), SS55 (Gary Cohen, Penn State University), Nanobodies described in this study are named Nb1_gbHSV - Nb17_gbHSV
Validation	Reference citations are available on the manufacturers websites for all antibodies.<br>rabbit anti-His6 antibody - https://www.abcam.com/en-us/products/primary-antibodies/6x-his-tag-antibody-ab9108?srsltid=AfmBOor8hj_ep9I-47nUJqsdXpKoUht2Kyg1PFrtymVxI0P4kLDBsjlc<br>anti-rabbit-HRP - https://www.sigmaaldrich.com/DE/de/product/sigma/a0545<br>mouse anti-GAPDH antibody - https://www.sigmaaldrich.com/DE/de/product/sigma/g8795<br>anti-mouse-HRP - https://www.sigmaaldrich.com/DE/de/product/mm/ap181p<br>SS55 - characterised in doi:10.1128/jvi.03200-13<br>Nanobodies Nb1_gbHSV - Nb4_gbHSV were further characterised and their ability to bind gB was validated by grating-coupled interferometry and cryoEM.

Eukaryotic cell lines

Policy information about cell lines and Sex and Gender in Research

Cell line source(s)	BHK-21 (C13), HEK293T & Vero (CCL-81) - all acquired from ATCC
Authentication	Authentication was only done via morphology and growth behaviour in the cell type specific media.
Mycoplasma contamination	All cell lines were regularly tested for mycoplasma contamination and all results were negative.
Commonly misidentified lines<br>(See ICLAC register)	None commonly misidentified cell lines were used.

Animals and other research organisms

Policy information about studies involving animals; ARRIVE guidelines recommended for reporting animal research, and Sex and Gender in Research

Laboratory animals	One female alpaca was used, kept at the Alpaca Facility of the Max Planck Institute for Multidisciplinary Sciences (Göttingen).
Wild animals	The study did not involve wild animals.
Reporting on sex	For generation of nanobodies one female alpaca was immunized.
Field-collected samples	The study did not involve samples collected from the field.
Ethics oversight	Immunizations and blood sampling of the alpaca has been approved by the animal welfare authority LAVES with the reference numbers 33.9-42502-05-13A351, 33.9-42502-05-17A220, and 33.19-42502-04-22-00210.

Note that full information on the approval of the study protocol must also be provided in the manuscript.

Plants

Seed stocks	n/a
Novel plant genotypes	n/a
Authentication	n/a
